# Carol of the Maison Dieu

**Published:** 1904-01-16

**Authors:** 


### Carol of the Maison Dieu.

[The exact date of a carol is seldom to be made out. The *Maison Dieu*, one of the largest hospitals in Paris, is of a great age: it was founded by Saint Louis, was nursed by a sisterhood, and Vesalius and Ambroise Paré were among its students in the earlier part of the sixteenth century. We can imagine them, with the sisters, going the round of the wards at Christmas-time, singing this interesting and comparatively unknown carol, which is now published for the first time in English in this Journal. The admixture of monkish Latin in each verse suggests a date prior to the Reformation. The metre of the carol in its present form has been adapted to that of the familiar English carol, *Good Christian men, rejoice*: or rather, to that of the older German form of that same carol, *In dulci jubilo*, which has a similar admixture of Latin.]

### THE CAROL.

### All Singing.

In all the town is joy to-day:
The rich are clad in fine array:
E'en the poor put on their best,
Whereby their gladness is confessed—
*Ubi vos, ibi nos*—
All the world on Christmas Day
Puts its grief away.

### Doctors and Students.

The wise men came from out the East,
That wisdom so might be increased;
Came to find the Word most wise
Hid behind a baby's eyes—
*Ubi Deus, ibi Meus*—
That was their exceeding prize,
Wisdom for the wise.

### Sisters and Nurses.

The shepherds came adown the hill,
With sturdy feet and right good will:
Came through all the night so wild,
To help the mother and the child—
*Ubi Crux, ibi Lux*—
Brought, to warm them, sheepskins rude,
Brought them fuel and food.

### All Singing.

The wise men, they had ease and wealth:
The shepherds, they had strength and health:
You, alas! lie here in pain,
And some may ne'er be whole again—
*Ubi vos, ibi nos*—
How shall we not sing in vain,
But turn your loss to gain?

### Doctors and Students.

To you, in God's Hostel, we come
That we may seek and find wisdom:
We, from your complaints, do tell
How to make your neighbours well—
*Ubi Lux, ibi Dux*—
All our wisdom is from you,
And the Word in you.

### Sisters and Nurses.

To you we bring your drink and food,
And pile your hearth with flaming wood,
Make your beds, and guard your sleep,
And you in all convenience keep—
*Ubi vos, ibi nos*—
Serve and help you day and night,
Through the dark to light.

### All Singing.

The wise men Eastward went away:
The shepherds did no longer stay:
We from you the Word receive,
We in you the Work retrieve—
*Ubi Meus, ibi Deus*—
All the world on Christmas Day
Puts its grief away.

